# Symptom Burden of Patients with Dry Eye Disease: A Four Domain Analysis

**DOI:** 10.1371/journal.pone.0082805

**Published:** 2013-12-13

**Authors:** Joelle A. Hallak, Sarmad Jassim, Vishakha Khanolkar, Sandeep Jain

**Affiliations:** 1 Corneal Neurobiology Laboratory, Department of Ophthalmology and Visual Sciences, University of Illinois at Chicago, College of Medicine, Chicago, Illinois, United States of America; 2 Department of Epidemiology and Biostatistics, University of Illinois at Chicago, School of Public Health, Chicago, Illinois, United States of America; University of Illinois at Chicago, United States of America

## Abstract

**Purpose:**

To determine which sensory (symptom persistence and intensity) and reactive (activity and affective interference) domains of symptom analysis are essential for assessing symptom burden in dry eye disease (DED) patients.

**Methods:**

A symptom domain tool was developed to investigate all four symptom domains in DED. In a cross-sectional pilot study, we administered the symptom burden tool and the Ocular Surface Disease Index (OSDI) questionnaire to 48 DED patients. Total and domain scores from the symptom burden tool and the OSDI were normalized to achieve comparability. Spearman correlation coefficients were calculated to measure the relationship between domains and subscales. Agreement between the symptom burden tool and OSDI was assessed by Bland-Altman plot. Assigned treatments were compared by symptom burden to determine whether treatment aggressiveness is linked to symptom intensity.

**Results:**

There was high agreement between the symptom burden tool and the OSDI. Symptom persistence had a stronger correlation with affective interference (r  =  0.62 for the symptom burden tool and r = 0.73 for the OSDI) than activity interference (r = 0.58 for the symptom burden tool and r = 0.60 for the OSDI). Symptom intensity correlated weakly with affective interference (r = 0.38) and activity interference (r = 0.37) in the symptom burden tool (OSDI does not have a subscale for intensity). In patients with equal persistence of symptoms, those having high symptom intensity were receiving more aggressive treatment (66.7%) than those with lower symptom intensity (33.3%).

**Conclusions:**

Persistence of symptoms correlates better with affective interference than activity interference. Intensity of symptoms may be important for treatment decisions.

## Introduction

Dry eye disease (DED) is a complex symptomatic disease with inexplicable clinical variations. With a prevalence ranging from 5% to over 35% at various ages [1], DED is one of the leading causes of patient visits to ophthalmologists and optometrists in the United States due to its debilitating symptoms [2], [3]. Several clinical tests are available to measure the aspects of DED. However, there is no gold standard for diagnosis, and clinicians rely on patient reported symptoms of ocular discomfort to make treatment decisions.

The reported symptoms of DED include pain, dryness, grittiness, itchiness, redness, burning or stinging, foreign body sensation, and light sensitivity. These symptoms have been reported to negatively impact the quality of life, with a greater risk of depression and anxiety for those with more symptoms [4], [5]. Given the variability of clinical tests, assessing DED symptoms in their entirety becomes fundamentally important to guide treatment decisions. In other chronic diseases, symptoms are thought of as a “burden,” and are measured in domains to encompass both the persistence and intensity of the symptoms and the patient’s perception of the impact of the symptoms [6], [7]. The total assessment of symptoms in similar domains is not often used in DED. While there are tools that measure the entire scope of DED, their utility is limited to clinical research. Developing a brief tool that comprehensively measures the symptom burden of DED without increasing respondent burden is needed for daily clinical use and diagnosis. A starting point is to adopt concepts used to measure symptoms in other diseases, and tailor them to symptoms of DED.

The method of domain assessment of symptoms is used in chronic diseases such as symptom control of cancer, especially when cure or remission is no longer possible [7]. Pain questionnaires, such as the Brief Pain Inventory (BPI), the Brief Fatigue Inventory (BFI), and the M. D. Anderson Symptom Inventory were developed to measure pain and discomfort. These tools are designed to assess symptoms in multiple dimensions and domains (7). The domains include intensity and severity (sensory dimension), and affective and activity interference (reactive dimension) [7], [8]. The rationale for use of a four domain tool is that it is specifically tailored to measuring multiple patient-reported symptoms and their impact. This applies very well to DED, given the underlying neurophysiological mechanisms of pain, and that DED is a chronic progressive disease. We therefore hypothesize that a more complete symptom assessment using 4 domains that characterize the “symptom burden” of DED will be more reflective of the disease, and will better indicate optimal treatment.

In this study, we developed a tool to investigate the four domain symptom burden of DED for ease of use in clinical settings, to determine the roles of symptom persistence and symptom intensity of DED, and their impact on activity and affective interference. We also performed a cross-sectional pilot study administering both the DED symptom burden tool and the Ocular Surface Disease Index (OSDI) questionnaire for cross comparison.

## Methods

Study approval was obtained from the Institutional Review Board of the University of Illinois at Chicago. Symptomatic patients with DED were enrolled and written informed consent was obtained from all patients after the nature and possible consequences of research were explained. Research was conducted in accordance with the requirements of the Health Insurance Portability and Accountability Act and tenets of the Declaration of Helsinki.

### Developing a Four Domain Symptom Burden Tool

Based on our findings from the literature regarding DED symptoms, a four domain DED symptom burden tool was developed adapting methods from well-established and validated symptom burden tools. For example, the affective interference domain included the same questions from the M.D. Anderson Symptom Questionnaire (mood, enjoyment of life, and social relations with others).

**Figure 1:**
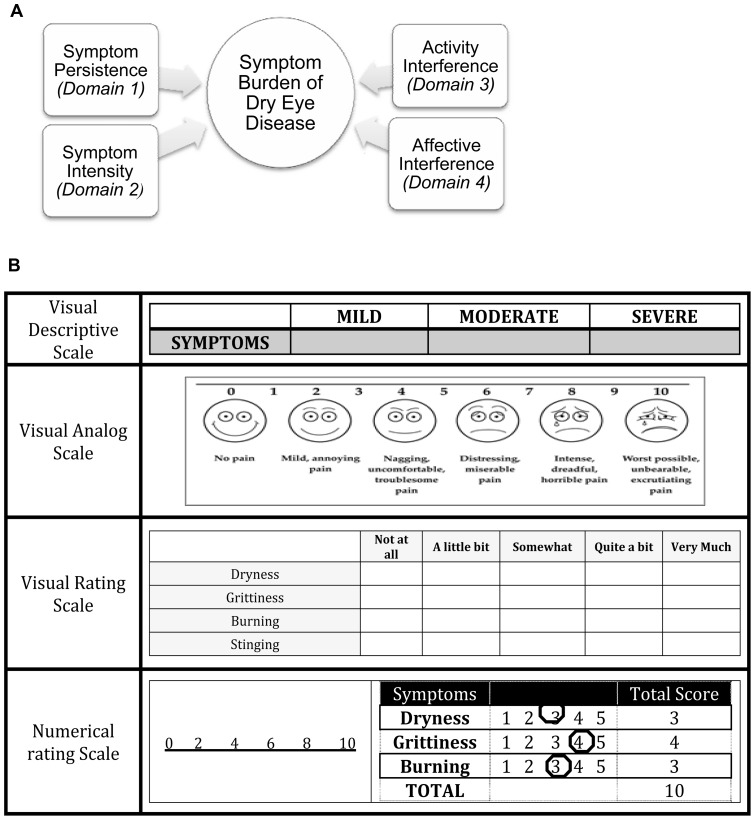
Symptom burden domains and measurement scales. (A). Domains of Symptom Burden of Dry Eye Disease. The symptom burden of dry eye disease is divided into two main dimensions: sensory dimension and reactive dimension. The sensory dimension is divided into two domains, symptom persistency and symptom intensity, while the reactive dimension is divided into activity interference and affective interference [Adapted from reference # 7]. (B). Scales for Measuring Symptoms. The visual analog and numerical scales are used to measure intensity of symptoms, whereas the visual rating scale is used to measure persistence of symptoms.

Classification into dimensions and domains: The two main dimensions assessed were sensory and reactive dimensions. Based on these dimensions the symptom burden was classified into four main domains (Figure 1A): (i) Sensory Dimensions – Symptom Persistence and Symptom Intensity, and (ii) Reactive Dimensions – Activity Interference and Affective Interference. Symptom persistence can be defined as the continuous occurrence of symptoms, whereas symptom intensity is the severity of symptoms. Activity interference is the effect of dry eye symptoms on day-to-day activities of an individual. Affective interference is the effect on the emotional and social wellbeing of an individual due to dry eye symptoms.

Scales used in domains: Various scales were used that organized symptoms into domains (Figure 1B). These scales can be described as follows: (i) Verbal Descriptive Scale (VDS), which classifies symptoms according to the methods of assessment that include measurements of mild/moderate/severe symptoms, (ii) Visual Analog Scale (VAS), a scale used frequently in the measurement of DED symptoms [8], VAS is used for describing pain that cannot be characterized by words with the use of visual images on a scale of one to ten, (iii) Numerical Rating Scale (NRS) classifies symptoms based on numerical scales such as 0 to 20 or 0 to 75, and is used as a mode of assessment of symptoms in dry eye studies, and (iv) Verbal Rating Score (VRS), which describes the occurrences of symptoms as none of the time, some of the time, most of the time, or all of the time.

**Figure 2:**
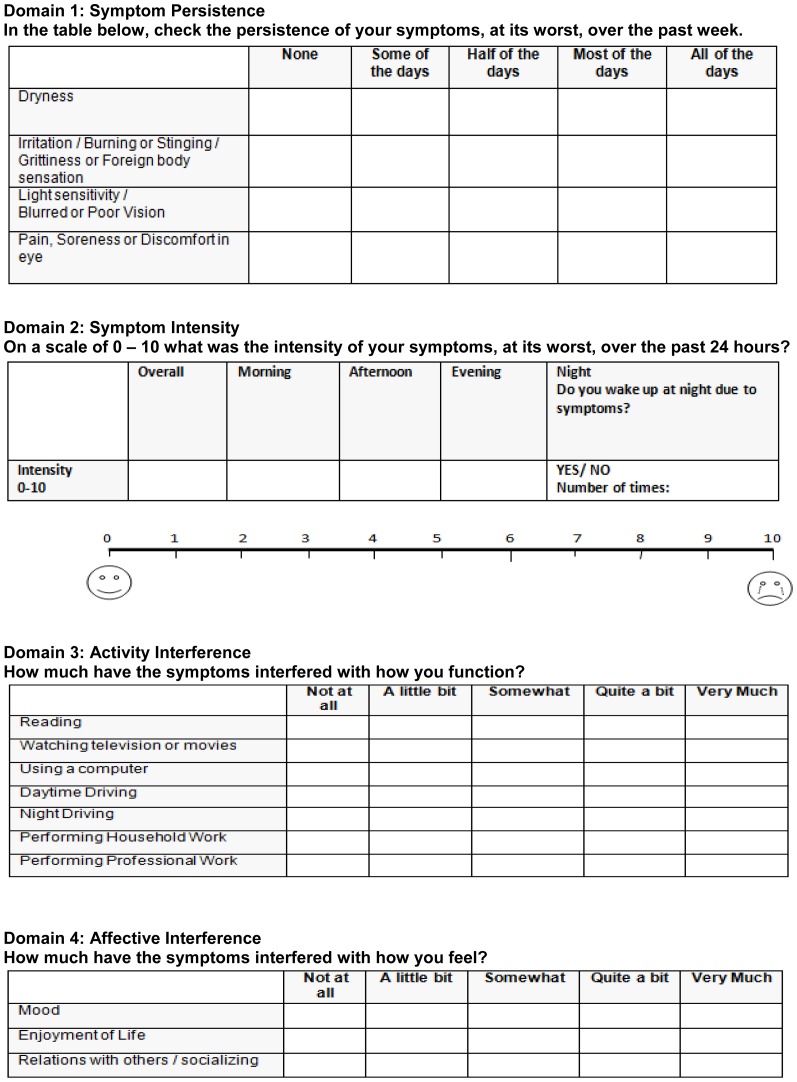
Dry Eye Symptom Burden Tool consisting of four domains: Symptom persistence, symptom intensity, activity interference, and affective interference.

The four-domain symptom burden tool that we developed is shown in Figure 2. A visual rating scale was used for the persistence, activity, and affective interference domains, and a combination of scales (visual analog and numerical) was used for the intensity domain. The visual analog scale is commonly used for assessing severity of symptoms (acuteness of pain) in a variety of settings. After generating the symptom burden tool, a cross-sectional pilot study was performed where the DED symptom burden tool and the Ocular Surface Disease Index (OSDI) questionnaire were administered to 48 patients.

### Data Collection and Patient Population

Patients were recruited from the Dry Eye Clinic of the Department of Ophthalmology and Visual Sciences, University of Illinois at Chicago. New and established patients, diagnosed with DED through the assessment of symptoms and clinical signs, were recruited over the course of a 6 month period, from October 2012 through March 2013. The symptom burden tool and the OSDI questionnaires were administered to patients in an interview during one visit. Subjects were asked about the persistence of their symptoms over the past week to minimize recall limitation.

Clinical examination included measuring tear production by the Schirmer’s test (without anesthesia) at 5 minutes, using Whatman filter strips #41 (Haag-Streit, Essex, UK). Severity of ocular surface disease was assessed using Rose Bengal dye. Saline moistened (1%) Rose Bengal-impregnated strips were used to instill the dye on the inferior palpebral conjunctiva, and scoring of corneal and conjunctival staining was performed by a slit lamp examination after 15 seconds.

The inclusion criteria were patients with Schirmer’s test results of < 10 mm in either eye and Rose bengal corneal and conjunctival staining of ≥ 1. Patients who were less than 18 years and women who were pregnant were excluded from the study.

To determine whether intensity of symptoms correlated with treatment decision, out of the 48 subjects included in the study, we randomly selected 9 pairs (18 patients), where each pair had equal symptom persistence scores but varying intensity scores. Prescribed treatments for each patient pair were collected. We scored each treatment option as either 1 point or 2 points as follows: Artificial tears (1 point); Restasis (1 point); Doxycycline/Erythromycin eye ointment (1 point); Steroids (2 points); therapeutic contact lens use (2 points); serum/DNase/other (2 points). Total treatment scores were then computed for each patient.

A weighted item response analysis was performed for the symptom burden tool: items from the persistence domain were summed and multiplied with the intensity, and the sum of activity and affective scores was then added to compute a total symptom burden score. Intensity scores were computed by multiplying the overall intensity with the number of times a patient reported waking up at night due to symptoms. The OSDI (index) score was calculated from OSDI item responses following standard procedures [9].

### Statistical Analysis

Items in each domain were summed to generate domain scores. Domain scores were then standardized by subtracting the mean from each individual score in each domain and dividing by the standard deviation to generate normalized comparative scores. Q-Q plots and Shapiro Wilk tests were run to determine whether the data is normally distributed. Inter-domain correlations were performed using the non-parametric Spearman test for each of the symptom burden and OSDI questionnaires, to determine whether persistence of symptoms with or without intensity correlated with activity and affective interference. Pearson correlation was not utilized because the data were not normally distributed; however fitted lines with scatter plots are shown for data representation. Cross domain and subscale correlations were also performed. Subscales A and B in the OSDI were considered to represent persistence of symptoms and activity interference, respectively.

To determine whether intensity of symptoms correlated with treatment decision, the total treatment score assigned to subjects in each pair were compared using a matched paired t-test.

Bland–Altman analyses were performed to determine agreement between normalized symptom burden scores and normalized OSDI scores. A range of agreement was defined as mean ± 2 SD. All analyses were performed using SAS software (SAS Institute Inc., Cary, NC) and STATA (StataCorp LP., College Station, TX) software. Confidence intervals at the 95% level were computed, and significance was determined if the interval did not include 0.

## Results

The patient population consisted of 32 females and 16 males with mean age of 52.8 years. Ten patients were diagnosed with auto-immune DED, 32 with non-autoimmune DED, and 7 with graft-versus-host disease related DED.

Within the symptom burden tool, higher correlations were observed between persistence of symptoms and affective interference than persistence of symptoms and activity interference (r  =  0.62; 95% CI [0.39, 0.77] versus r  =  0.58; 95% CI [0.35, 0.75]) (Table 1). The correlation between the OSDI persistence subscale and affective interference domain in the symptom burden tool was r  =  0.73; 95% CI [0.56, 0.84] (Table 1). Multiplying the persistence of symptoms with the intensity did not improve the correlation in the symptom burden tool for activity interference (r  =  0.54) and for affective interference (r  =  0.56). Correlations between intensity of symptoms alone, and activity and affective interference were low, r  =  0.37 [95% CI 0.08, 0.60] and r  =  0.38 [95% CI 0.09, 0.60], respectively, with the symptom burden tool.

**Table 1:** Spearman Correlation between and across domains in the Symptom Burden Tool (SB) and the OSDI questionnaire

Domain r [95% CI]	PersistenceSB	IntensitySB	Persistence x Intensity (SB)	Activity Interference SB	Affective Interference SB	Persistence OSDI	Activity OSDI
Persistence SB	1.00	0.27 [−0.03,0.53]	0.75 [0.59,0.86]	0.58 [0.35,0.75]	0.62 [0.39,0.77]	0.76 [0.60,0.86]	0.53 [0.29,0.72]
Intensity SB	0.27 [−0.03,0.53]	1.00	0.81 [0.67,0.89]	0.37 [0.08,0.60]	0.38 [0.09,0.60]	0.47 [0.21,0.67]	0.26 [−0.03,0.52]
Persistence x Intensity	0.75 [0.59,0.86]	0.81 [0.67,0.89]	1.00	0.54 [0.3,0.73]	0.56 [0.32,0.74]	0.76 [0.59,0.86]	0.55 [0.30,0.73]
Activity Interference SB	0.58 [0.35,0.75]	0.37 [0.08,0.60]	0.54 [0.3,0.73]	1.00	0.68 [0.48,0.81]	0.60 [0.37,0.76]	0.79 [0.65,0.88]
Affective Interference SB	0.62 [0.39,0.77]	0.38 [0.09,0.60]	0.56 [0.32,0.74]	0.68 [0.48,0.81]	1.00	0.73 [0.56,0.84]	0.64 [0.43,0.79]
Persistence OSDI	0.76 [0.60,0.86]	0.47 [0.21,0.67]	0.76 [0.59,0.86]	0.60 [0.37,0.76]	0.73 [0.56,0.84]	1.00	0.55 [0.30,0.72]
Activity OSDI	0.53 [0.29,0.72]	0.26 [−0.03,0.52]	0.55 [0.30,0.73]	0.79 [0.65,0.88]	0.64 [0.43,0.79]	0.55 [0.30,0.72]	1.00

SB: Symptom Burden; OSDI: Ocular Surface Disease Index.

**Figure 3:**
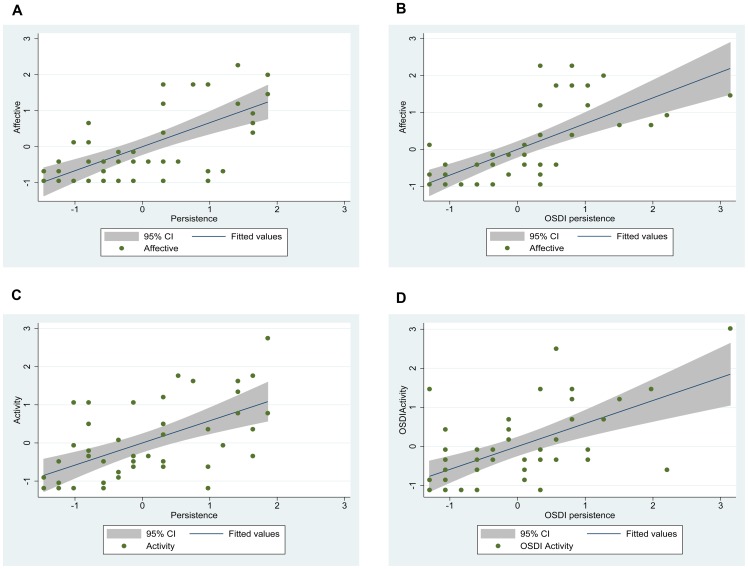
Scatter Plots Between Persistence Domain in the Symptom Burden Tool or Persistence Subscale in the OSDI Questionnaire versus Affective and Activity Interference. Persistence calculated from the OSDI subscale A plotted with affective interference shows the highest linear agreement with a coefficient of determination R^2^ of 0.49. The coefficient is comparable to that of persistence from the symptom burden tool and its relationship with affective interference (R^2^  =  0.44). Activity interference showed lower coefficients of determination R^2^ of 0.34 and 0.35 for symptom persistence calculated from the symptom burden tool and symptom persistence from the OSDI subscale.

Figures 3A−D show the scatter plots, with the best fitted lines and the 95% confidence interval (CI), between scores of persistence of symptoms and affective interference and persistence of symptoms and activity interference with the symptom burden tool and OSDI questionnaires. The best fitted linear relationship is shown between persistence of symptoms as measured by the OSDI subscale A and affective interference as measured with the symptom burden tool (R^2^  =  0.49) (Figure 3B).

**Figure 4:**
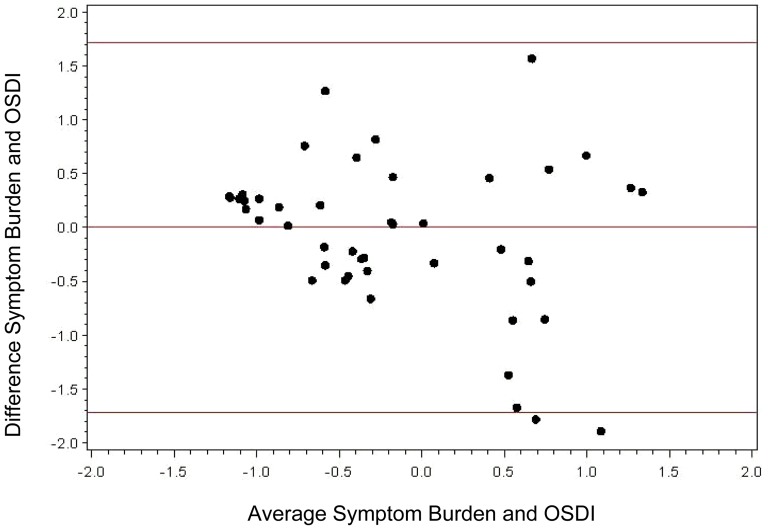
Bland-Altman Plot for assessing symptoms of DED with the symptom burden tool and OSDI. The 95% confidence limits of agreement between the two methods ranged from −1.7 to 1.7. As shown, 46 out of 48 scores are within the 95% confidence interval, indicating good agreement between the OSDI and Symptom Burden Scores.

Bland-Altman analysis showed that most values are between +/− 2 SD of the mean difference between the symptom burden tool scores and OSDI scores. The 95% confidence limits of agreement between the two methods ranged from −1.7 to 1.7, depicting good agreement between OSDI total scores and the symptom burden total scores (Figure 4).

With regards to the effect of intensity of symptoms on treatment decision, six out of the nine pairs (66.7%) had patients reporting high intensity symptom burden 33.3% (3 of 9 pairs) were patients reporting low intensity symptom burden (A difference between high and low intensity of > 4 was used as a cutoff point). The mean difference in symptom burden score between high and low intensity was 10.56 (SD = 5.68) (14.89 high vs. 4.33 low, *p < 0.0001*). The mean treatment score for patients with high intensity symptoms was 6.33 (SD = 1.32) and 3.78 (SD = 1.92) for patients with low intensity symptoms, for a mean difference in treatment score of 2.56 (SD  =  2.96, *p = 0.03*). There were three patients with low intensity who received aggressive treatment, were mainly due to either neuropathic pain (one patient with post-LASIK DED reported high intensity), or due to a marginal difference in intensity with equal persistence.

## Discussion

Our study revealed three main findings: (i) Affective interference correlates more strongly with persistence of DED symptoms, (ii) the synergistic effect of intensity of symptoms with persistence of symptoms did not increase the correlation, and (iii) intensity of symptoms may play a role in treatment decisions. The persistence of symptoms in the OSDI did show a moderate correlation with activity interference. However, activity interference alone may not be a good index of the overall suffering of DED patients because it overlooks the emotional and psychological aspects (affective interference). Our results showed that the persistence of symptoms in OSDI actually correlates better with affective interference in the symptom burden tool rather than with activity interference. Therefore, our pilot data makes a case for including affective interference in tools which assess DED symptoms. Evaluating the “symptom burden” of DED in its entirety will allow us to better delineate responses to treatments.

DED has been shown to negatively impact the quality of life of patients, including general quality of life and vision-related quality of life [10], [11]. Furthermore, DED has been shown to be correlated with anxiety and depression [4], [5], [12]. The negative impact on the quality of life is mainly due to the progression of dry eye symptoms, creating a complex situation that interferes with daily activities and the emotional state of DED patients [1]. The OSDI activity interference has been utilized by studies to measure the impact of DED on quality of life [13]. However, the OSDI does not include an affective component. It is a disease specific questionnaire which includes three subscales or domains: ocular discomfort (OSDI symptoms, equivalent to persistence), functioning (OSDI function, equivalent to activity interference), and environmental triggers (OSDI triggers) [9], [14]. In addition to the OSDI, studies have also used more generic instruments such as the National Eye Institute Visual Function Questionnaire (NEI-VFQ) to measure the quality of life of DED patients [13], [15]. The NEI-VFQ is a 25 item questionnaire with 11 subscales/domains, of which mental functioning is one. Vitale et al compared the use of the NEI-VFQ and the OSDI to examine the associations between vision-targeted health related quality of life and ocular surface parameters in patients with Sjogren’s syndrome [13]. They examined subscale/domain correlations between the two instruments. Associations between OSDI and NEI-VFQ subscales were modest and the report concluded that both instruments were similar in their ability to measure the impact of Sjogren’s syndrome-related dry eye on vision-targeted health related quality of life [13]. Li et al have simultaneously used both the OSDI and NEI-VFQ instruments to measure the quality of life [12], and more recently a new instrument known as the Impact of Dry Eye on Everyday Life (IDEEL) has been developed [16]. The IDEEL questionnaire includes 57 items that assess dry eye impact in three modules: symptom-bother, impact on daily life, and dry eye treatment satisfaction. The impact on daily life module included an emotional aspect. While the IDEEL was described as the only comprehensive questionnaire that assesses the entire scope of dry eye on patient outcomes, it is more useful in research settings as its regular clinical utility is limited by the time required to administer the questionnaire. Abetz et al do mention the reduction of items and the use of specific, but not necessarily all modules, to assess dry eye related quality of life [16].

The concept of symptom assessment of dry eye has been used elsewhere. Schaumberg et al developed and evaluated a short questionnaire based on a visual analog scale called the “Symptom Assessment iN Dry Eye (SANDE)” to quantify the frequency and severity of DED. While this instrument exhibited good reliability, it did not measure the symptom burden of DED in its entirety. In this study, we developed a tool adapting a variety of scales (visual analog scale, visual rating scale, and numerical rating scale) to measure the entire symptom burden of DED in domains used in other chronic studies that deemed to be necessary components to measure the impact of symptoms on quality of life. We believe that persistence and intensity of DED symptoms affect daily activities and the “mood” of individuals. However, our results show that intensity of symptoms did not correlate with activity and affective interference, whereas the persistence of symptoms showed much higher correlations, especially with affective interference. The importance of measuring the impact of DED symptoms on affective interference is consistent with recent studies showing an association between depression, anxiety, and DED [4], [5], [17].^7^

To further understand the role of intensity of symptoms, we determined whether intensity of symptoms would correlate with physician treatment of choice (aggressive versus non-aggressive). Our results showed that, irrespective of clinical signs, the majority of patients reporting more intense symptoms received aggressive treatments, whereas patients reporting low symptom intensity received less aggressive treatments. Physicians rely upon symptom analysis to make treatment decisions. The more symptomatic a patient is during a clinical visit, the more aggressive treatment he/she will receive. It becomes fundamentally important to analyze symptoms reliably and in their totality to guide treatment decisions.

The problems in evaluating efficacy of treatment in DED are related to incomplete understanding of symptom burden analysis. Traditional therapies for DED replace or conserve the patient’s tears without correcting the underlying disease process. These include tear replacement by topical artificial tears and punctal occlusion to prevent the drainage of natural or artificial tears [18]. The development of pharmacological therapies has been limited by our incomplete understanding of the mechanism, pathogenesis, and clinical manifestation of DED. Whether treatment is helpful or not is based on improvements in signs and symptoms. However there is a well-established disconnect between signs and symptoms [10], [19], [20]. The disconnect makes it difficult to determine whether the treatment is efficient. In addition, recent outcome studies and reviews on dry eye therapies have shown that dry eye treatment needs to be tailored to the type and severity of dry eye disease [21]. This can only be done by effectively developing a multi-symptom patient-reported outcome tool for DED. Dry eye symptoms can persist for years and may worsen over time. Thus, there is a need to collectively assess the symptoms of dry eye and measure its symptom burden.

The symptom burden tool for DED, developed in our study, provides an efficient and easy method to measure the impact of symptom persistence and intensity on activity and affective interference and treatment decisions, respectively. There are several limitations to this study including the assessment of symptoms at one time point only and the small sample size. This is a pilot study and results cannot be broadly generalized. Studies with a larger sample size, in this population as well as other dry eye population groups, are required to further determine the content, construct and criterion validity of the symptom burden tool. Specifically, a predictive validity study is required to measure the association between the burden domains with one or two outcome measures over time, such as changes in symptoms over time or the effects of treatment. Additionally, prospective studies where the symptom burden is measured at several time points are needed to measure the reliability of the symptom burden tool. Despite these limitations, we believe that adding an affective component to standardized questionnaires for DED, such as the OSDI, may allow us to determine the effect of persistence of DED symptoms on psychological and social wellbeing. Measuring the intensity of symptoms will allow us to further understand treatment responses and develop treatment decisions.
